# Residual Notch Stress Intensity Factors in Welded Joints Evaluated by 3D Numerical Simulations of Arc Welding Processes

**DOI:** 10.3390/ma14040812

**Published:** 2021-02-08

**Authors:** Alberto Campagnolo, Paolo Ferro, Luca Romanin, Giovanni Meneghetti

**Affiliations:** 1 Department of Industrial Engineering, University of Padova, 35131 Padova, Italy; alberto.campagnolo@unipd.it; 2 Department of Management and Engineering, University of Padova, 36100 Vicenza, Italy; paolo.ferro@unipd.it (P.F.); luca.romanin@phd.unipd.it (L.R.)

**Keywords:** coarse mesh, finite element analysis, peak stress method, residual notch stress intensity factor, residual stress, welding simulation

## Abstract

Approaches based on calculating Residual Notch Stress Intensity Factors (R-NSIFs) assume the weld toe to be a sharp V-notch that gives rise to a residual singular stress distribution close to the weld toe. Once R-NSIFs are determined, they might be included in local fatigue criteria for the structural strength assessment of welded joints based on NSIFs due to external cyclic loading. However, the numerical calculation of R-NSIFs through finite element (FE) simulations of the welding process requires extremely refined meshes to properly capture the residual stress singularity. In this context, the Peak Stress Method (PSM) has recently been adopted to estimate R-NSIFs due to residual stresses by means of coarse meshes of 2D 4-node plane or 3D 8-node brick elements. The aim of this work is to investigate the applicability of the PSM to estimate R-NSIFs in a butt-welded joint using coarse meshes of 3D 10-node tetra elements. The R-NSIF distribution at the weld toe line is estimated by applying the PSM to coarse meshes of 3D 10-node tetra elements, and the results are in agreement with those obtained using 3D 8-node brick elements. Accordingly, the PSM based on tetra elements further enhances the rapid estimation of R-NSIFs using coarse meshes and could be effective in analyzing complex 3D joint geometries.

## 1. Introduction

The development of fatigue approaches that account for the effect of residual stresses induced by the arc welding process is a topic of increasing interest not only in the scientific literature but also in the industrial context [1,2,3,4,5]. For this purpose, numerical simulations aimed at predicting residual stress distributions due to the welding process [6,7,8,9,10,11,12,13,14,15,16] should be as reliable as possible, but they must also be rapid to meet industrial needs. In this context, Okano et al. [17] recently performed a finite element (FE) simulation by coupling weld mechanics and the arc plasma process and obtained a good agreement between numerically calculated residual stress fields and experimental measurements carried out using the X-ray diffraction method.

**Figure 1:**
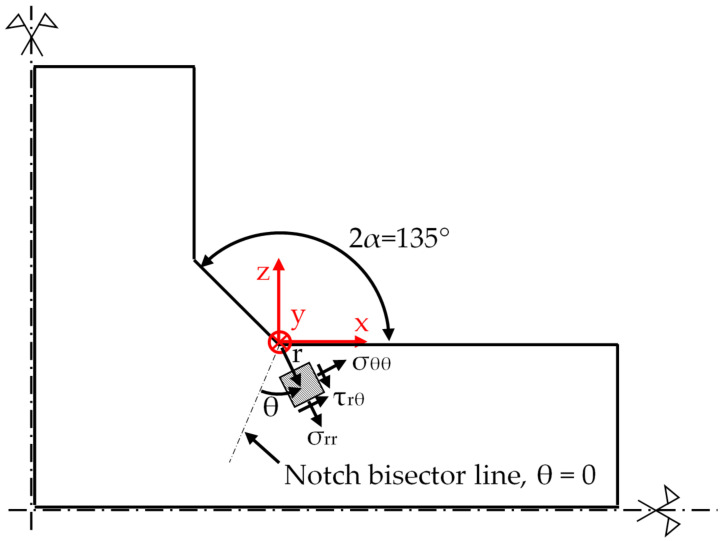
Geometrical assumptions to define the Residual Notch Stress Intensity Factor (R-NSIF) at the weld toe of a cruciform full-penetration welded joint: the sharp V-notch opening angle 2α is typically 135°, while the tip radius is null. The polar reference system is centered at the weld toe and residual stress components are highlighted.

Several approaches have been proposed in the technical literature to analytically represent residual stress fields near the weld toe of welded structures in order to include their effects in fatigue criteria. Among these methods, the singular linear elastic stress field derived by Williams [18] and the elastic-plastic stress field proposed by Hutchinson, Rice, and Rosengren (HRR solution) [19,20] have been widely adopted for the consideration of welding process parameters and boundary conditions. Both Williams and HRR solutions are applicable to the analysis of the local residual stress field if the weld toe profile is assumed to be a sharp V-notch with a null tip radius, i.e., the “worst case” condition [21], while the notch opening angle is typically 2α = 135° (see Figure 1). By adopting these geometrical assumptions and the Williams solution [18], a Residual Notch Stress Intensity Factor (R-NSIF) can be defined for mode I according to Equation (1). K_I_ quantifies the intensity of the residual stress field near the weld toe, and it represents a sound residual stress parameter to be included in local criteria for the fatigue assessment of welded joints [22,23,24].

(1) $$K_{I}=\sqrt{2\pi }\cdot \underset{r\to 0}{\lim}\left[{\left(\sigma_{\theta \theta }\right)}_{\theta =0}\times r^{1-\lambda_{1}}\right]$$

where the linear elastic local stress component σ_θθ_ of the residual stress field is calculated near the weld toe (r→0) and along the notch bisector line (θ = 0), as sketched in Figure 1, while the parameter (1 − λ_1_) is the stress singularity exponent [18], which equals 0.326 for 2α = 135°.

Calculating the R-NSIF according to Equation (1) in accordance with Gross and Mendelson [25], requires (i) evaluating the whole asymptotic stress distribution along the V-notch bisector line by means of welding process simulations with the adoption of extremely refined meshes (with the minimum element size equal to approximately 10^−5^ mm, [22,26], and (ii) postprocessing the calculated set of stress-distance data in order to derive the R-NSIF value. It should be noted that welding numerical simulations are intrinsically nonlinear and transient; therefore, they are extremely demanding in terms of computational time, especially when 3D joint geometries and/or multipass 3D welded structures are under investigation [27,28,29,30,31]. For these reasons, 2D FE models are typically employed in engineering practice to calculate R-NSIFs [22,23].

Rapid techniques that are able to estimate the intensity of residual stress fields in welded structures could be effective in introducing the R-NSIF approach in an industrial context owing to the considerable reduction of the computational time. Among available techniques, the Peak Stress Method (PSM) allows for the estimation of the NSIFs from singular, linear elastic peak stresses calculated at the tip of sharp V-notches using coarse meshes with a uniform element size, which must comply with a proper range of applicability [32,33,34,35]. The PSM originates from the method formulated by Nisitani and Teranishi [36,37] for rapidly calculating the mode I stress intensity factor (SIF) at the tip of a circumferential crack propagating from an ellipsoidal cavity. Since it was originated, the PSM has been theoretically justified and extended to estimate the NSIF of sharp V-notches under either mode I [32,33] or mode III [38] loadings and the SIF of cracks under mode II [39] loading. The PSM is advantageous as compared with calculating NSIFs according to their analytical definition, Equation (1), since (i) coarser mesh patterns (having an element size that is often several orders of magnitude larger) can be used and (ii) the calculation of only the singular, linear elastic peak stresses at the notch tip is sufficient; in other words, the set of stress–distance data required to compute the NSIFs from Equation (1) is not necessary.

In previous papers [40,41], the rapid estimation of the mode I R-NSIF of a butt-welded joint has been performed by applying the PSM to 2D coarse meshes of 4-node plane elements. Subsequently, the PSM was extended to rapidly calculate the R-NSIF from 3D coarse meshes of 8-node brick elements [42]. In the present work, the PSM is extended to estimate the R-NSIF from 3D coarse meshes of 10-node tetra elements. This approach further enhances the rapid estimation of the R-NSIF, since mesh patterns of tetra elements can be generated by free meshing even complex 3D joint geometries; in contrast, the PSM based on brick elements requires a regular mesh pattern, at least locally near the weld toe.

## 2. The Peak Stress Method (PSM)

**Figure 2:**
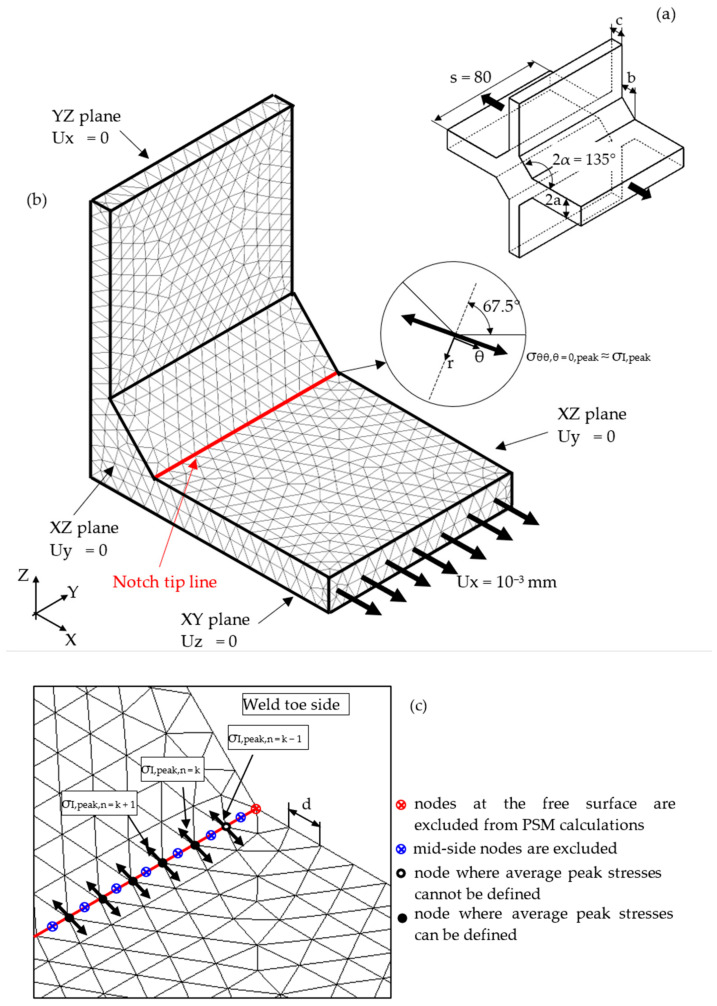
FE model to apply the Peak Stress Method (PSM) according to Equation (2) at the weld toe of a full-penetration cruciform welded joint (**a**) under axial loading using 3D 10-node tetra elements (**b**). Considered case: 2a = 13 mm, b = 10 mm, c = 8 mm, and 2α = 135°. Free mesh pattern of 10-node tetra elements of Ansys^®^ code with element size d = 3 mm. Applied load: displacement U_x_ = 10^−3^ mm. (**c**) Nodes where peak stresses can be computed and adopted to estimate the NSIF.

The PSM is a numerical technique that allows for a rapid estimation of the NSIF parameter K_I_, previously defined in Equation (1). The opening peak stress, σ_θθ,θ = 0,peak_, calculated at the V-notch tip by a linear elastic FE analysis with a coarse mesh, as illustrated in Figure 2, is adopted to estimate K_I_ by means of Equation (2) [32]:

(2) $$K_{I}≅K_{FE}^{*}\cdot \sigma_{\theta \theta ,\theta =0,\mathrm{peak}}\times d^{1-\lambda_{I}}$$

where d is the average element size adopted to generate the mesh pattern. When analyzing the weld toe, it is worth noting that the opening peak stress, σ_θθ,θ = 0,peak_, approximately corresponds to the maximum principal stress σ_I,peak_, which is easier to calculate since it does not require a polar reference system aligned with the notch bisector line. The coefficient K^*^_FE_ depends on the (i) element type and integration scheme, (ii) pattern of finite elements, and (iii) procedure employed by the FE code to extrapolate nodal stresses [43]. The parameter K^*^_FE_ has been calibrated using several 2D and 3D element types and commercial FE software under the conditions discussed in the relevant literature [32,33,42,43,44], to which the reader is referred. A state-of-the-art review on the PSM and its applications to the fatigue strength assessment of welded joints has recently been published [35].

### 2.1. Two-Dimensional 4-Node Plane Elements

The 2D PSM was originally calibrated in [32] using 4-node quadrilateral plane elements of the Ansys^®^ 2020 R2 (ANSYS Inc., Canonsburg, PA, USA) element library (PLANE 182 with K-option 1 set to 3, which corresponds to a ‘simple enhanced strain’ 2 × 2 integration scheme), and the resulting coefficient was K^*^_FE_ = 1.38 ± 3%. Subsequently, the parameter K^*^_FE_ was calibrated with 2D finite elements available in six commercial numerical codes: Abaqus^®^, Straus7^®^, MSC^®^ Patran/Nastran, LUSAS^®^, HyperMesh/OptiStruct/HyperView^®^, and HyperMesh/Ls-Dyna/HyperView^®^, again obtaining K^*^_FE_ = 1.38 ± 5% [43]_._ Recently, the coefficient K^*^_FE_ was calibrated in [40] by adopting 2D 4-node plane elements available in Sysweld^®^ (2004 with a 2 × 2 full-integration scheme), and the resulting coefficient was K^*^_FE_ = 1.64 ± 5%. It is worth noting that previous values of K^*^_FE_ are valid only if the adopted mesh pattern satisfies the following conditions: the number of elements that share the node located at the notch tip must be 4 when 2α ≤ 90° and 2 when 2α > 90° (e.g., at the weld toe, 2α ≅ 135°).

### 2.2. Three-Dimensional 8-Node Brick Elements

The PSM was then extended to analyze 3D FE models; therefore, 8-node brick elements of the Ansys^®^ element library were calibrated (SOLID 185 with K-option 2 set to 3, corresponding to a “simple enhanced strain” 2 × 2 integration scheme), and it again resulted in K^*^_FE_ = 1.38 ± 3% [33], i.e., the same value calibrated for 2D 4-node plane elements. In [42], the coefficient K^*^_FE_ previously calibrated for 2D 4-node plane elements of Sysweld^®^, i.e., K^*^_FE_ = 1.64 ± 5%, was also successfully verified for 3D 8-node brick elements available in Sysweld^®^ (3008 with a full-integration scheme corresponding to 8 integration points). It should be noted that the same conditions for the mesh pattern, previously described for 2D 4-node plane elements, must also be respected to apply the PSM using 3D 8-node brick elements. However, it might be difficult to apply the PSM to 3D welded structures using brick elements since the required regular mesh pattern cannot be generated in complex joint geometries. In these cases, first, a main model of the whole geometry must be free-meshed by adopting 10-node tetra elements; after that, a submodel of the local geometry at the weld toe can be analyzed by the PSM using brick elements.

### 2.3. Three-Dimensional 10-Node Tetra Elements

**Figure 3:**
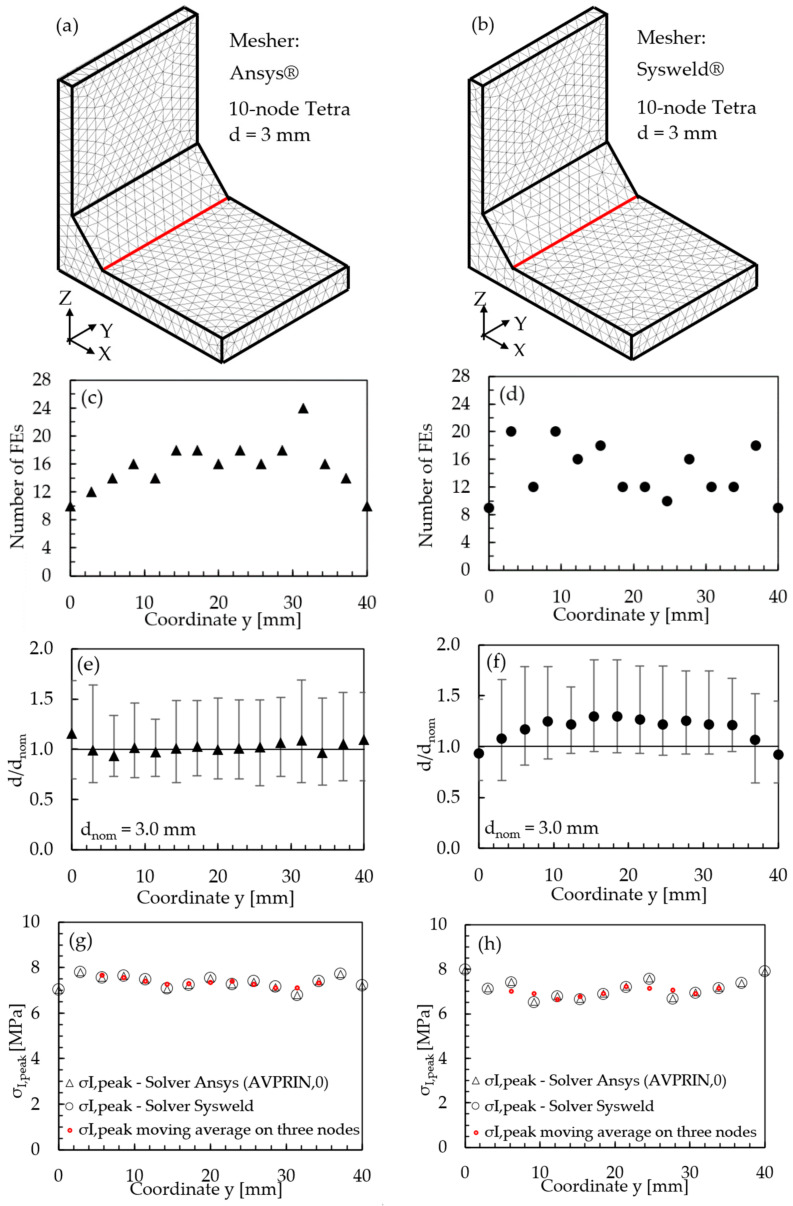
FE models to apply the PSM to the welded joint of Figure 2. Free mesh pattern of 10-node tetra elements with nominal element size d = 3 mm and generated in (**a**) Ansys^®^ and (**b**) Sysweld^®^ code. (**c**,**d**) Number of finite elements that share each vertex node at the weld toe. (**e**,**f**) Normalized size of finite elements that share each vertex node at the weld toe. (**g**,**h**) Comparison of peak stress distributions calculated by Ansys^®^ and Sysweld^®^ along the weld toe line of the FE models reported in (**a**,**b**), respectively.

To speed up the application of the PSM to 3D FE models, the coefficient K^*^_FE_ was calibrated in [44] using 10-node tetra elements of the Ansys^®^ element library (SOLID 187 with 4 integration points). Accordingly, the PSM can be directly applied to the free-meshed main model (see example in Figure 2); therefore, a submodel that has a regular mesh pattern is rendered unnecessary. However, tetra mesh patterns are typically irregular; i.e., each node located at the notch tip line can be shared by a different number of elements (see Figure 3c,d) with significantly different sizes (see Figure 3e,f) and shapes (see [35]).

As a consequence, the peak stress can vary along the notch tip line even when a constant NSIF exists (see example in Figure 3g,h). To overcome this issue, an average peak stress was proposed in [45] to smoothen the peak stress distribution along the notch tip line. In detail, a moving average peak stress calculated at three adjacent vertex nodes was defined according to the following expression (which is relevant to the case of node n = k):

(3) $${\bar{\sigma }}_{I,\mathrm{peak},n\;=\;k}={\left.\frac{\sigma_{I,\mathrm{peak},n\;=\;k-1}+\sigma_{I,\mathrm{peak},n\;=\;k}+\sigma_{I,\mathrm{peak},n\;=\;k+1}}{3}\right|}_{n\;=\;\mathrm{node}}$$

Accordingly, the coefficient K^*^_FE_ was calibrated for 10-node tetra elements (SOLID 187 of Ansys^®^) by inserting the average peak stress defined in Equation (3), i.e., ${\bar{\sigma }}_{I,\mathrm{peak}}$, instead of the peak stress σ_I,peak_ into Equation (2) [45], and it resulted in K^*^_FE_ = 1.05 ± 15% for 0 ≤ 2α ≤ 120° and K^*^_FE_ = 1.21 ± 10% for 2α = 135°. It is worth noting that previous values of K^*^_FE_ are valid only if the following conditions are fulfilled (see Figure 2, [35]): (i) peak stresses acting on nodes at a free surface of the welded joint must be neglected since a distorted mesh pattern can affect their values and (ii) only peak stresses calculated at vertex nodes of tetra elements must be employed in Equation (3): i.e., peak stresses at mid-side nodes must be neglected.

Finally, it is worth noting that the parameter K^*^_FE_ has never been calibrated by adopting 3D 10-node tetra elements available in Sysweld^®^. On the other hand, calibration would further enhance the rapid estimation of the R-NSIF since mesh patterns of tetra elements can be generated by directly free meshing a volume: this approach is more rapid and more efficient for discretizing complex 3D joint geometries, as compared with the meshing technique required to generate a regular mesh pattern according to the PSM based on brick elements.

## 3. Application of the 3D PSM Based on 10-Node Tetra Elements in the Sysweld^®^ Environment

In order to apply the 3D PSM based on 10-node tetra elements to FE analyses performed in Sysweld^®^, first, calibration of the coefficient K^*^_FE_ is necessary. To do this, several 3D notch problems under pure mode I loading should be analyzed by calculating (i) the average peak stress ${\bar{\sigma }}_{I,\mathrm{peak}}$ (Equation (3)) using coarse mesh patterns with element size d and (ii) the exact value of the NSIF K_I_ (Equation (1)) by adopting extremely refined FE meshes. After that, for each considered geometrical, meshing and loading configuration, the parameter K^*^_FE_ can be evaluated from Equation (2), re-arranged as follows:

(4) $$K_{FE}^{*}=\frac{K_{I}}{{\bar{\sigma }}_{I,\mathrm{peak}}\times d^{1-\lambda_{I}}}$$

To fully calibrate the parameter K^*^_FE_ while taking into account the variability of the results due to different geometrical configurations or mesh patterns, the number of analyzed 3D FE models should be in the range of 50–100, according to previous calibrations [32,38,39,44]. This task was performed in [40] for calibrating the 2D PSM based on 4-node plane elements available in the Sysweld^®^ environment. However, it is evident that a full calibration based on several 3D FE analyses would be a time-consuming activity; therefore, in the present work, only a comparison between mesh patterns and peak stresses calculated with Ansys^®^ and Sysweld^®^ was carried out in order to check if the coefficient K^*^_FE_ previously calibrated in Ansys^®^ code could also be adopted in the Sysweld^®^ environment.

The full-penetration cruciform welded joint under axial loading, shown in Figure 2 and having thickness 2a = 13 mm and notch opening angle at the weld toe 2α = 135°, was considered as a case study. Free mesh patterns of 10-node tetra elements with a nominal element size d = 3 mm were generated in Ansys^®^ and Sysweld^®^ codes, as shown in Figure 3a,b, respectively. Qualitatively comparing Figure 3a with Figure 3b suggests that very similar mesh patterns are generated by Ansys^®^ and Sysweld^®^; however, a more detailed analysis was carried out to perform a quantitative comparison. Figure 3c,d report the number of finite elements that share each vertex node along the weld toe line and show that it ranges between 10 and 24 for Ansys^®^ mesh and between 9 and 20 for Sysweld^®^ mesh, with a less clear trend of scattered results provided by Sysweld^®^. Figure 3e,f reports the size, i.e., the length of the tetrahedron edges, of finite elements that share each vertex node at the weld toe line and show that the average element size closely matches the nominal one, d_nom_ = 3 mm, with a variability in the range of 0.64·d_nom_–1.69·d_nom_ for Ansys^®^ mesh; for Sysweld^®^, the average element size is always larger than the nominal one, and it falls in the range of 0.64·d_nom_–1.85·d_nom_.

After that, the peak stress distributions calculated by Ansys^®^ and Sysweld^®^ codes were compared by excluding the effect of the FE mesh. To do this, the free mesh pattern of Figure 3a, generated in Ansys^®^, was imported into the Sysweld^®^ environment and, vice versa, the free mesh pattern of Figure 3b, generated in Sysweld^®^, was imported into Ansys^®^ code. Therefore, identical mesh patterns were used with both numerical software. For the element type and formulations, the 10-node tetra element corresponds to

- Ansys^®^: SOLID 187 with 4 integration points, no other formulations being available;
- Sysweld^®^: 3010 with 4 integration points and two possible integration schemes, i.e., reduced (INTEG = 2 in Sysweld^®^) or full (INTEG = 3 in Sysweld^®^), which, however, provide the same results.

The material properties of a structural steel, i.e., Young’s modulus E = 206,000 MPa and Poisson’s ratio ν = 0.3, were assumed. Taking advantage of the symmetry conditions, only one-eighth of the joint was analyzed. Moreover, a plane strain condition was simulated by constraining the out-of-plane displacement U_y_ (which translates in a null strain component, ε_y_ = 0) according to Figure 2. A nodal displacement Ux equal to 10^−3^ mm was applied at the nodes of the boundary of the loaded plate to simulate the axial loading.

It is worth mentioning that identical nodal stress extrapolation and averaging criteria were adopted in Ansys^®^ and in Sysweld^®^ codes. In detail, nodal stresses in the element were extrapolated from the integration points of FEs, and then nodal stress components were calculated by averaging the nodal stresses of the elements that share that node. Principal stresses were evaluated from averaged nodal stress components, which corresponds to the option “average from components” (AVPRIN,0 setting) in Ansys^®^ code.

The results in terms of peak stresses, i.e., the maximum principal stress σ_I,peak_, calculated along the weld toe line (y-direction) are reported in Figure 3g,h, which illustrate a perfect match of the 10-node tetra elements available in Ansys^®^ and Sysweld^®^ codes. Moreover, the differences in the mesh patterns generated by Ansys^®^ and Sysweld^®^ highlighted in Figure 3c–f only slightly affect the peak stress distributions: indeed, Figure 3g,h show that the mesh pattern generated by Ansys^®^ provides a peak stress in the range of 6.79–7.79 MPa, while that generated by Sysweld^®^ produces a peak stress in the range of 7–8 MPa. Therefore, it is reasonable to assume that the coefficient K^*^_FE_ = 1.21 that has been previously calibrated in Ansys^®^ is also valid in Sysweld^®^ [44] for V-notches with the opening angle 2α = 135° as the weld toe. Indeed, the comparison reported in Figure 3 demonstrates that Ansys^®^ and Sysweld^®^ have the same (i) element type and integration scheme for 10-node tetra and (ii) procedure to extrapolate stresses at nodes; on the other hand, they generate different mesh patterns, which affect the output peak stress distribution but only slightly affect the average peak stress values calculated according to Equation (3).

## 4. Rapid R-NSIF Estimation Using the PSM

**Figure 4:**
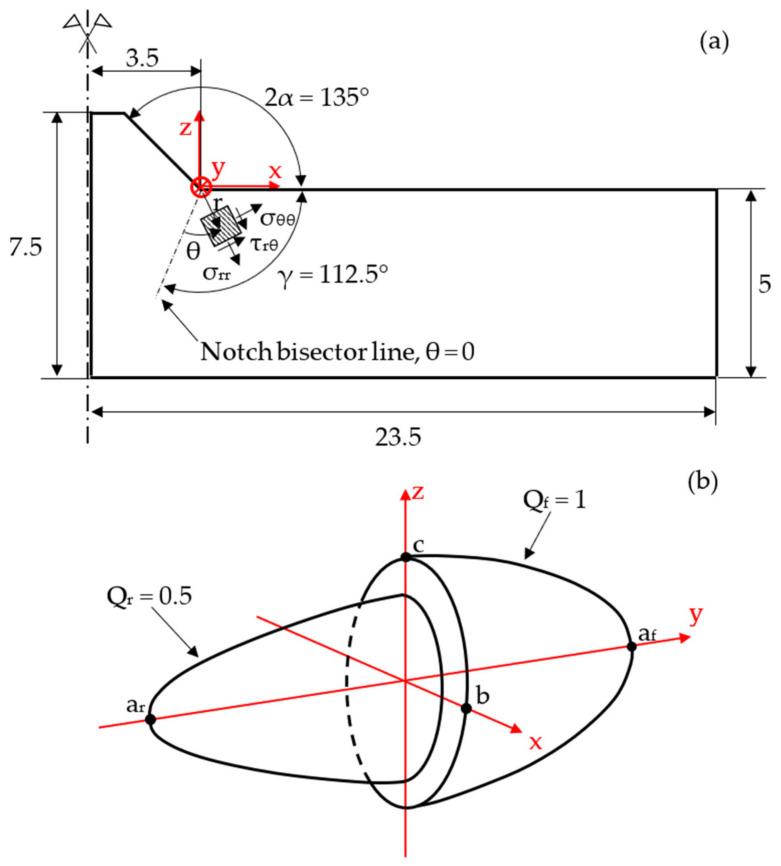
(**a**) Geometry of the analyzed butt-welded joint. (**b**) Double ellipsoid heat source. Reproduced with permission from [42] (Copyright 2019 Elsevier).

Sequentially coupled thermometallurgical and mechanical welding FE simulations were performed using Sysweld^®^ code to calculate the R-NSIFs generated in a butt-welded joint via the welding process. Figure 4a reports the longitudinal cross-section of the analyzed butt-welded joint, with the width equal to 30 mm in the y-direction and the notch opening angle 2α = 135° at the weld toe. The considered material is ASTM SA 516 carbon steel, whose metallurgical, thermal, and mechanical properties, depending on the phases (i.e., austenite, ferrite, pearlite, bainite, and martensite) and temperature, have been taken from [28]. Goldak’s power density distribution function developed for the arc welding process simulation, as illustrated in Equation (5) [46], was adopted to simulate the welding heat source. The double ellipsoid power density distribution shape according to Goldak’s Equation (5) is sketched in Figure 4b.

(5) $$\left\{\begin{array}{c} Q_{F}=Q_{f}\exp\left(\frac{-x^{2}}{b^{2}}\right)\exp\left(\frac{-y^{2}}{a_{f}^{2}}\right)\exp\left(\frac{-z^{2}}{c^{2}}\right)\;\mathrm{source}\;\mathrm{front}\\ Q_{R}=Q_{r}\exp\left(\frac{-x^{2}}{b^{2}}\right)\exp\left(\frac{-y^{2}}{a_{r}^{2}}\right)\exp\left(\frac{-z^{2}}{c^{2}}\right)\;\mathrm{source}\;\mathrm{rear} \end{array}\right.$$

In the above expressions, Q_F_ and Q_R_ represent the frontal and rear power density, respectively; Q_f_ and Q_r_ are the maximum frontal and rear power density, respectively, while, a_f_, a_r_, b, and c are Gaussian parameters of Goldak’s heat source [46], as described in Figure 4. Goldak’s heat source is centered on the symmetry plane, over the upper surface. All heat source parameters adopted in the FE analyses are summarized in Table 1.

All metallurgical phase transformations that occur during heating and cooling, as well as their effects on residual stress distribution, i.e., volume change and transformation plasticity, were taken into account, as reported in [28,47,48,49]. In the mechanical simulation, a dedicated function was employed to set the mechanical properties of the material to zero in all nodes where the computed temperature was higher than the melting point.

Heat loss boundary conditions were applied at the external surfaces of the plates to be joined:

- radiative heat loss according to the Stefan–Boltzmann law, i.e., $q_{r}=\varepsilon_{r}\sigma_{r}\left(T^{4}-T_{0}^{4}\right)$, where ε_r_ = 0.7 is the emissivity of the material surface and σ_r_ is the Stefan–Boltzmann constant;
- convective heat loss assuming a convective heat transfer coefficient equal to 25 W/m^2^K, as suggested by the Sysweld^®^ manual and in agreement with a high air-flow assumption [50].

**Figure 5:**
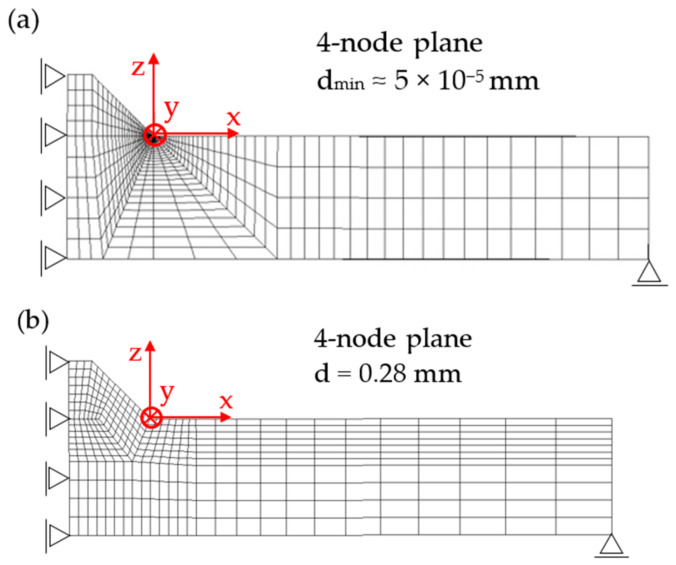

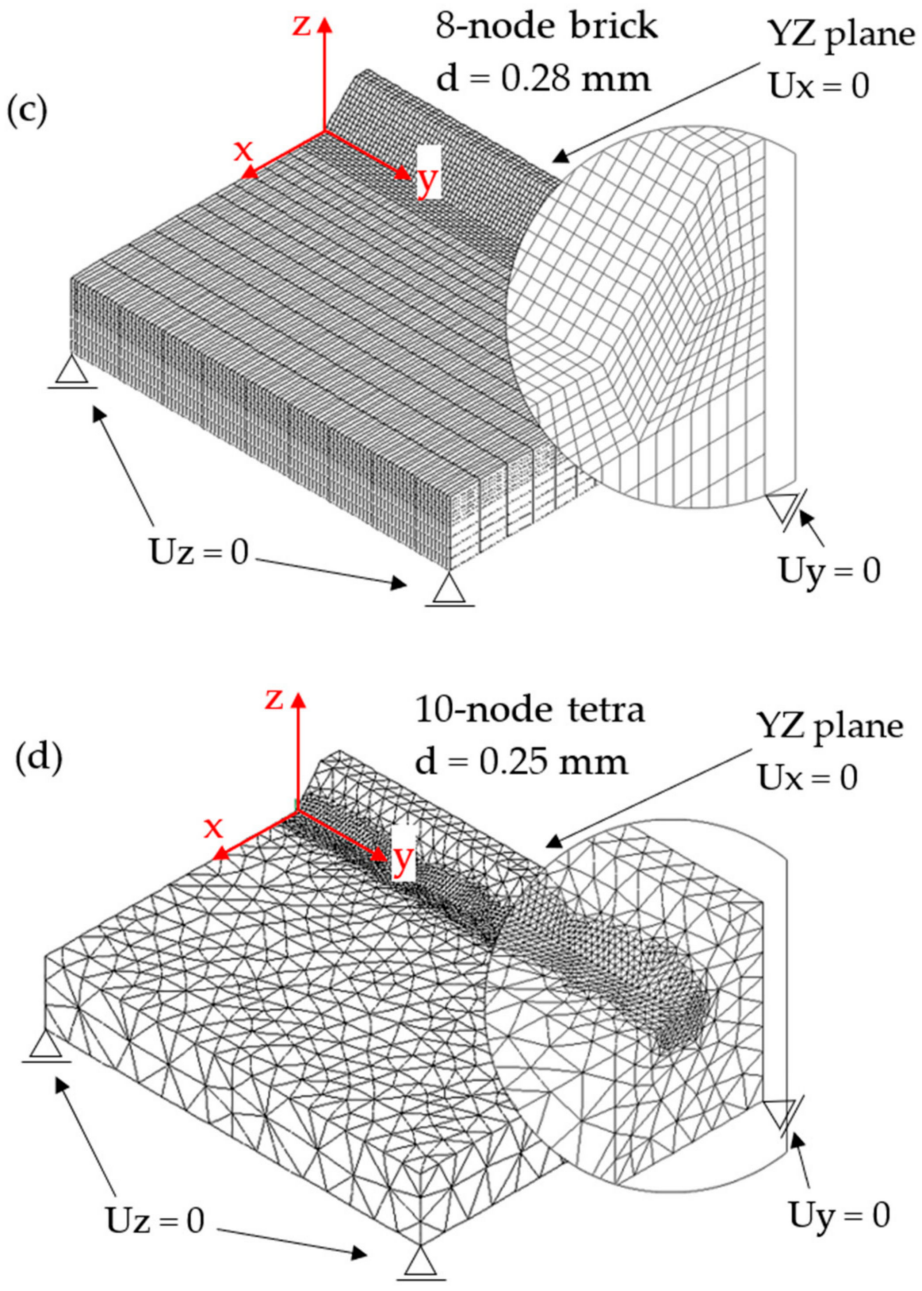
(**a**) Refined FE model: 2D mapped mesh with 4-node plane elements. Coarse FE models: (**b**) 2D mapped mesh with 4-node plane elements; (**c**) 3D mapped-mesh with 8-node brick elements, obtained by extruding the 2D mesh; and (**d**) 3D free mesh with 10-node tetra elements.

The symmetry of both the geometry and the thermal load allowed for the simulation of only one-half of the joint (see Figure 4a). Moreover, the constraint conditions sketched in Figure 5 were adopted during and after the welding process to prevent rigid body motion.

For comparison purposes, first, a 2D FE analysis was performed by adopting 4-node plane elements available in Sysweld^®^ (2004 with a 2 × 2 full-integration scheme) under generalized plane strain conditions. To calculate the R-NSIF from the residual singular stress distributions, a very refined mapped mesh with a minimum element size equal to about 5 × 10^−5^ mm was defined near the weld toe (Figure 5a), according to Lazzarin and Tovo [26].

After that, several FE analyses were carried out by adopting coarse meshes according to the PSM:

- A 2D FE model of 4-node plane elements available in Sysweld^®^ (2004 with a 2 × 2 full-integration scheme) under generalized plane strain conditions. A coarse mapped mesh with element size d = 0.28 mm was generated near the weld toe (Figure 5b).
- A 3D FE model of 8-node brick elements available in Sysweld^®^ (3008 with a full-integration scheme corresponding to 8 integration points). The 3D mesh pattern (Figure 5c) was generated by extruding the 2D FE mapped mesh (Figure 5b) along the y-direction by adopting a step size equal to d = 0.28 mm.
- A 3D FE model of 10-node tetra elements available in Sysweld^®^ (3010 with a full-integration scheme corresponding to 4 integration points). The 3D mesh pattern (Figure 5d) was generated by directly free meshing the 3D volume of the analyzed butt-welded joint.

It should be noted that the results obtained from the FE models reported in Figure 5a–c were originally presented in [40,42], respectively, and they are recalled here for comparison purposes. On the other hand, the FE model of Figure 5d and its results are presented for the first time in the present paper.

## 5. Results and Discussion

**Figure 6:**
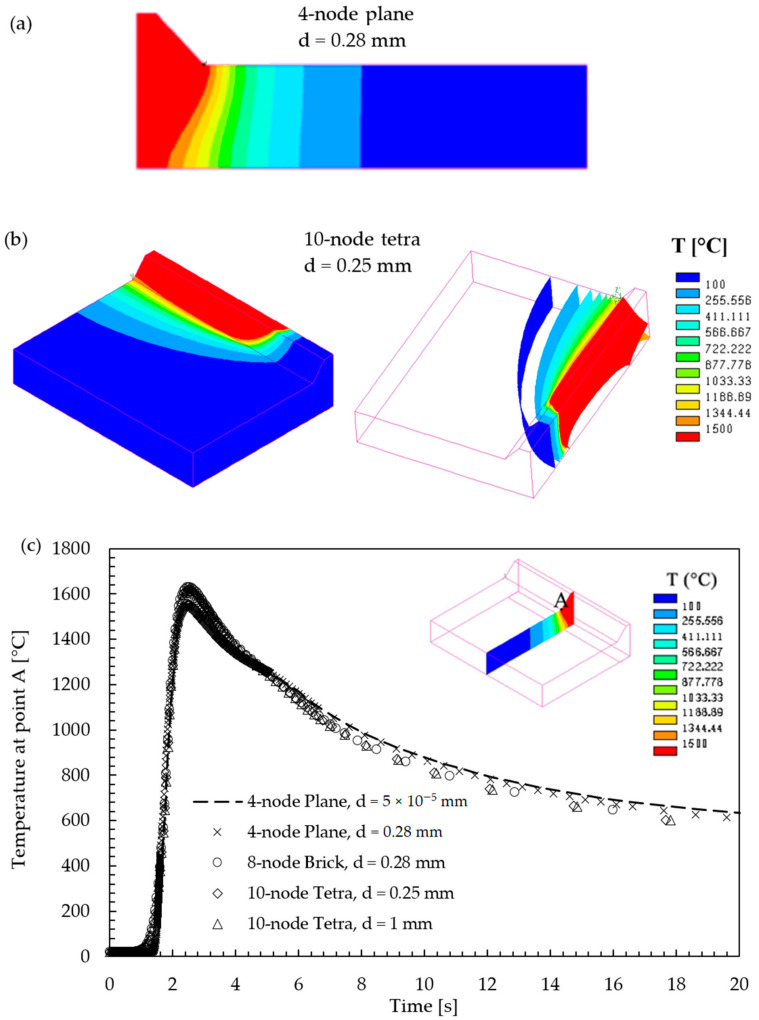
Comparison between thermal results from 2D and 3D models: snapshots captured when the heat source is traveling over the cross-section (**a**) or the welding line (**b**); the fusion zone shapes (in red) obtained by 2D and 3D FE models, respectively, are highlighted; and (**c**) temperature history at node A as a function of the adopted element type and average element size d.

First, the 2D and 3D FE models were compared in terms of temperature history results. Figure 6a,b compares the temperature plots and the fusion zone shapes (in red) obtained as outputs from 2D 4-node plane and 3D 10-node tetra FE models, respectively. The figures illustrate that the 3D model accounts for a thermal gradient along the welding y-direction, which, in contrast, the 2D model cannot simulate. Despite this, Figure 6c shows that a good correlation exists between the temperature history at node A, which is located at the half-width longitudinal section of the joint, as calculated by either 2D or 3D FE models. Node A is of interest since the following results show that the maximum R-NSIF value occurs at the half-width longitudinal section of the joint, and therefore, values of R-NSIF calculated by the different FE models are compared at that location. Moreover, it is worth noting that Figure 6c also includes the temperature history calculated by a 3D 10-node tetra FE model, discretized with a coarser mesh, with element size d = 1 mm near the weld toe, as is discussed in the following Sections.

After verifying the convergence of the thermometallurgical results for all analyzed FE models, the mechanical results were compared. 

**Figure 7:**
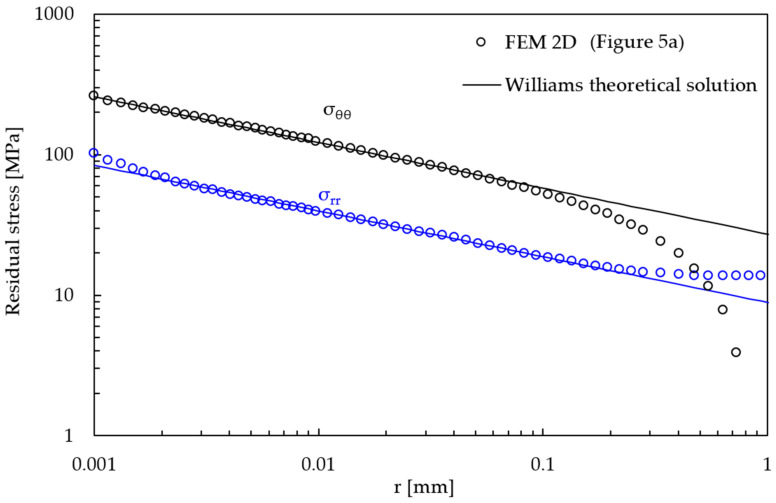
Asymptotic residual stress field obtained from the 2D FE model with extremely refined mesh (Figure 5a).

The residual singular stress fields near the weld toe were calculated along the notch bisector line (θ = 0 according to Figure 1) from the 2D FE model that has a very refined mapped mesh (Figure 5a). The obtained results in terms of stress components σ_θθ_ and σ_rr_ as a function of the radial distance *r* from the weld toe are reported in Figure 7 and compared with the theoretical linear elastic solutions that have a stress singularity (1 − λ_I_) = 0.326, according to Williams [18]. Figure 7 shows that a deviation between the calculated residual singular stress components and the theoretical solution according to Williams exists at a large distance from the weld toe, i.e., for high values of *r*. This is due to the fact that the analytical solution based on the R-NSIF K_1_ is valid only in a local region near the weld toe, where the residual stress field is governed by the leading order term, i.e., K_1_. To represent the whole residual stress distribution, including in regions far from the singularity point, higher order nonsingular stress terms beyond K_1_ are necessary. By postprocessing the results of Figure 7 according to Equation (1) [25], a value of R-NSIF equal to 68.2 MPa·mm^0.326^ was obtained in [40].

**Figure 8:**
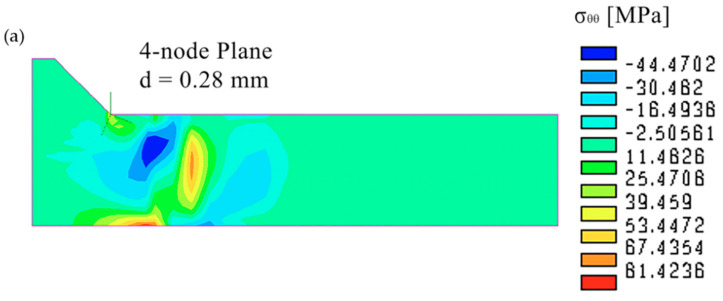

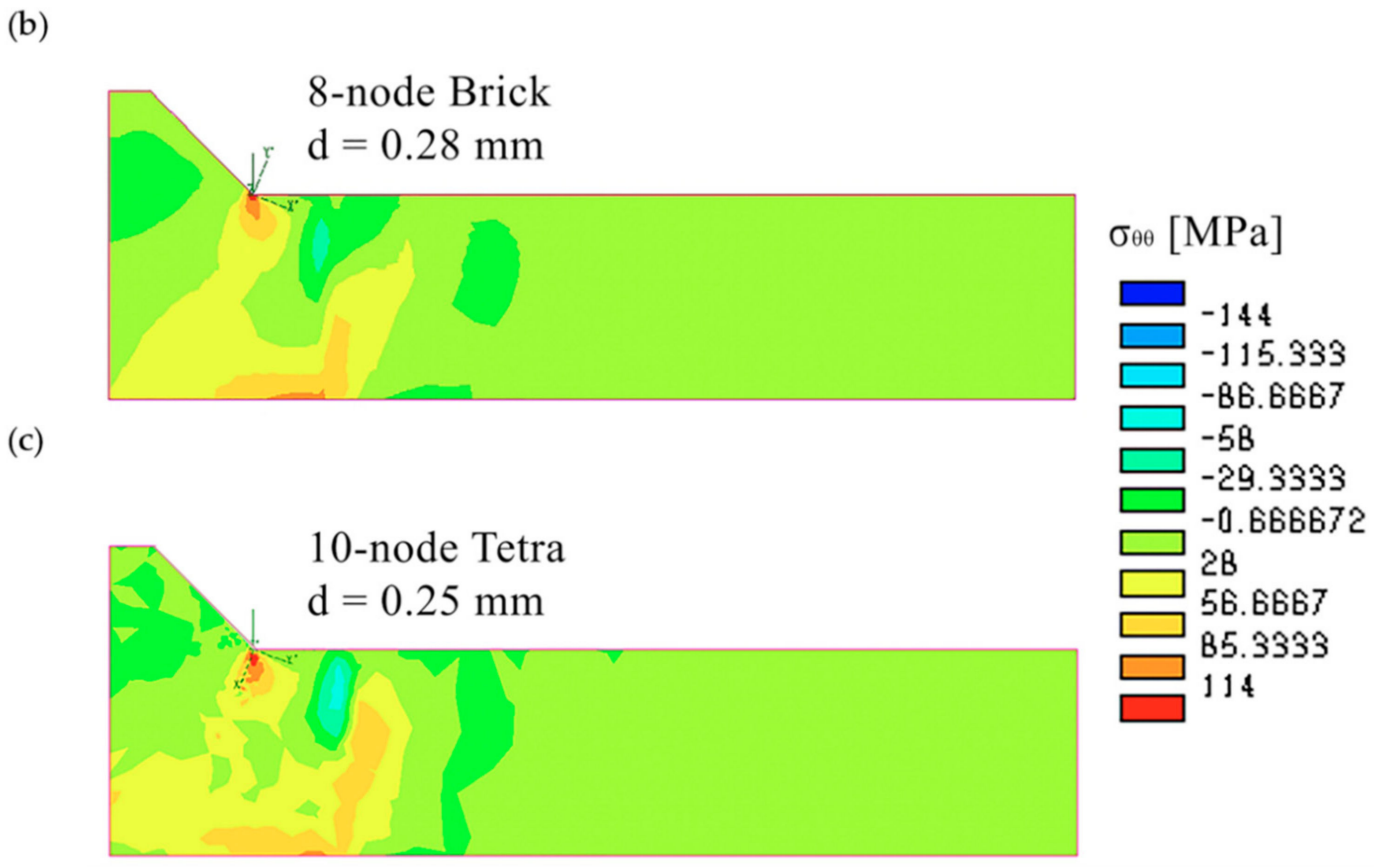
Comparison between the residual stress plots of the σ_θθ_ component calculated by FE models with coarse meshes of (**a**) 2D 4-plane elements, (**b**) 3D 8-node brick elements, and (**c**) 3D 10-node tetra elements. For the 3D models, the plots refer to the half-width longitudinal section.

Figure 8 compares the plots of the residual stress component σ_θθ_ calculated by FE models with coarse meshes (Figure 5b–d); the results of 3D models refer to the half-width longitudinal section, where the plane strain conditions are better matched and therefore comparable to those of the 2D model. A general similarity of the residual stress distribution plots is observed, but the numerical results are different, especially between 2D and 3D FE analyses. This is due to the effect of the thermal gradient along the welding y-direction on the resulting residual stress distribution, which is not accounted for by the 2D FE model, as previously observed in the comparison of Figure 6a,b.

After that, the PSM was applied to rapidly estimate the R-NSIF by postprocessing the results obtained from FE models with coarse meshes reported in Figure 5b–d:

- Two-dimensional 4-node plane elements (Figure 5b): The R-NSIF K_I_ was estimated by substituting the coefficient K^*^_FE_ = 1.64 [40], the opening peak stress calculated at the weld toe σ_I,peak_ = 63.02 MPa, the average element size d = 0.28 mm, and the stress singularity exponent (1 − λ_I_) = 0.326 in Equation (2). The resulting R-NSIF K_I_ is equal to 68.25 MPa·mm^0.326^, as shown in Equation (6) [40]. It is worth noting that the value obtained by the 2D PSM coincides with that calculated by applying Equation (1) to the results of the FE model with extremely refined mesh (Figure 5a).
  (6) $$K_{I}≅K_{FE}^{*}\times \sigma_{I,\mathrm{peak}}\times d^{1-\lambda_{I}}=1.64\cdot 63.02\;\mathrm{MPa}\times {\left(0.28\;\mathrm{mm}\right)}^{0.326}=68.25\;\mathrm{MPa}\times \;{\mathrm{mm}}^{0.326}$$
- Three-dimensional 8-node brick elements (Figure 5c): The R-NSIF was estimated by adopting the values of K^*^_FE_ = 1.64 [40,42] and average element size d = 0.28 mm, as shown in Equation (7), which highlights that, in the case of 3D FE models, a distribution of the R-NSIF K_I_ can be calculated as a function of the y-coordinate.
  (7) $$K_{I}(y)≅K_{FE}^{*}\times \sigma_{I,\mathrm{peak}}(y)\times d^{1-\lambda_{I}}=1.64\cdot \sigma_{I,\mathrm{peak}}(y)\times {\left(0.28\;\mathrm{mm}\right)}^{0.326}$$
  For the half-width longitudinal section of the 3D model, where the plane strain conditions are better matched, the resulting R-NSIF K_I_ is equal to 153.01 MPa·mm^0.326^ [42].
- Three-dimensional 10-node tetra elements (Figure 5d): The R-NSIF K_I_ was estimated by substituting the coefficient K^*^_FE_ = 1.21, the average element size d = 0.25 mm, and the stress singularity exponent (1 − λ_I_) = 0.326 in Equation (2), as shown in Equation (8). The R-NSIF K_I_ is proportional to the average opening peak stress (Equation (3)) calculated at the weld toe, which, in turn, depends on the y-coordinate:
  (8) $$K_{I}(y)≅K_{FE}^{*}\times {\bar{\sigma }}_{I,\mathrm{peak}}(y)\times d^{1-\lambda_{I}}=1.21\cdot {\bar{\sigma }}_{I,\mathrm{peak}}(y)\times {\left(0.25\;\mathrm{mm}\right)}^{0.326}$$

For the half-width longitudinal section of the 3D model, the resulting R-NSIF K_I_ is equal to 151.36 MPa·mm^0.326^, which is in perfect agreement with the value previously calculated by 3D 8-node brick elements.

Finally, the obtained results, along with details of FE simulations, are summarized in Table 2. It is worth noting that the R-NSIF values estimated by the PSM in the half-width longitudinal section of the 3D FE models are about 125% higher (153 MPa·mm^0.326^ instead of 68 MPa·mm^0.326^) than those resulting from the application of either the PSM or R-NSIF definition, Equation (1), to 2D FE models under generalized plane strain conditions. In Ref. [42], this deviation was attributed to the effect of the thermal gradient along the welding y-direction on the resulting residual stress distribution, which intrinsically cannot be accounted for in the 2D FE model.

**Figure 9:**
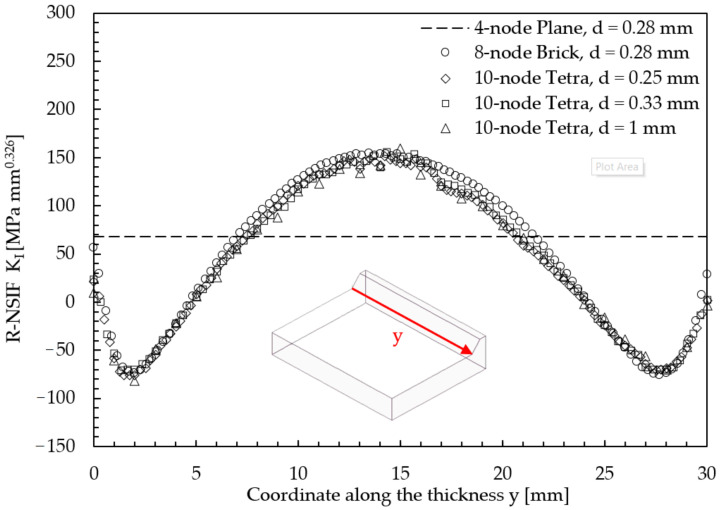
Comparison between R-NSIF distributions as a function of the element type and average element size d. The considered FE mesh patterns are reported in Table 3.

Using previous PSM-based expressions, i.e., Equations (7) and (8), the R-NSIF K_I_ can be plotted as a function of the welding y-direction, as shown in Figure 9, which reveals that (i) the maximum value of the R-NSIF occurs near the half-width longitudinal section of the joint and (ii) a constant R-NSIF distribution along the weld toe line is not obtained due to the reduced joint length.

Finally, it is interesting to compare R-NSIF K_I_ values that are estimated by adopting 3D 10-node tetra elements but by varying the average element size d, as highlighted in Table 3. Figure 9 compares the R-NSIF distributions and shows that for an element size d between 0.25 and 1 mm, the results obtained by applying the 3D PSM based on tetra elements appear to be mesh-insensitive. It is worth noting that the coarsest mesh of 10-node tetra elements considered in Figure 9, i.e., that with d = 1 mm, also provides thermal results that agree with those calculated by more refined 2D or 3D FE mesh patterns, as previously highlighted in Figure 6c. For comparison purposes, Figure 9 also includes results obtained by applying the PSM to coarse mesh of either 2D plane or 3D brick elements, as previously obtained in [40,42]. It is worth noting that when applying the PSM to rapidly estimate R-NSIF values, the maximum element size that can be adopted to generate the FE mesh depends not only on the geometrical size of the joint, which is the case for structural FE analyses to estimate the NSIF values [35], but also on the convergence of the results obtained from the thermal simulation [40,42]. Indeed, the results of mechanical FE analyses, such those reported in Figure 9, are reliable only if the thermal results are mesh-insensitive, as previously demonstrated for the same mesh patterns in Figure 6c.

## 6. Conclusions

In the present work, the Residual Notch Stress Intensity Factors (R-NSIFs) of a butt-welded joint were rapidly estimated by applying, for the first time, the Peak Stress Method to welding numerical models, where coarse meshes of 3D 10-node tetra elements were adopted. The following conclusions can be drawn:

- The through-the-thickness distribution of the R-NSIF estimated by applying the PSM to coarse meshes of 3D 10-node tetra elements is in agreement with results obtained in a previous work that adopted coarse meshes of 3D 8-node brick.
- 3D FE models account for the effect of the thermal gradient along the welding direction on the resulting residual stress distribution, which intrinsically cannot be accounted for in the 2D FE model.
- The applicability of the PSM to 3D coarse meshes of tetra elements further enhances the rapid estimation of the R-NSIFs; in fact, mesh patterns of tetra elements can be generated by directly free meshing the joint volume, which is more rapid and more efficient for discretizing complex 3D joint geometries, as compared with the meshing technique required to generate a regular mesh pattern according to the PSM based on brick elements.
- Finally, the PSM based on 3D coarse mesh patterns of tetra elements allows for a more detailed investigation of the residual stress distribution along the welding line and its influence on the fatigue strength behavior of welded joints through local approaches.

## Figures and Tables

**Table 1:** Goldak’s heat source parameters (see Figure 4b).

Q_f_ (W/mm^3^)	Q_r_ (W/mm^3^)	a_f_ (mm)	a_r_ (mm)	b (mm)	c (mm)	v (mm/s)
550	450	0.5	3	0.5	3.5	10

**Table 2:** Residual Notch Stress Intensity Factor (R-NSIF) values obtained by means of 2D and 3D FE models. The table includes also the central processing unit (CPU) time for each analysis.

FE Mesh	Minimum FE Size (mm)	CPU Time (min)	K_I_ (MPa mm^0.326^)
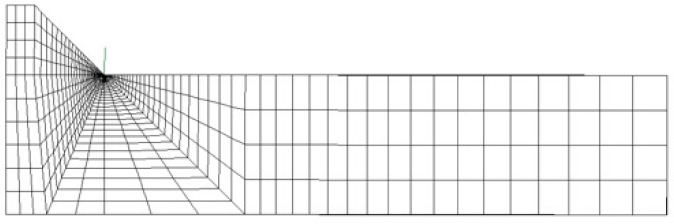	10^−5^ #FE 1782 #nodes 1681	33	68.20
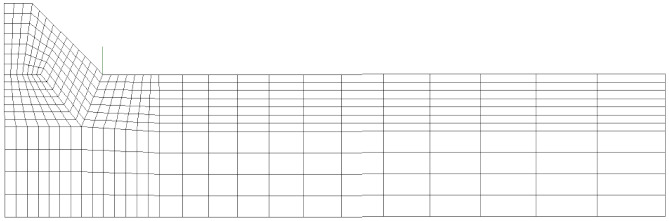	0.28 #FE 763 #nodes 766	8	68.25
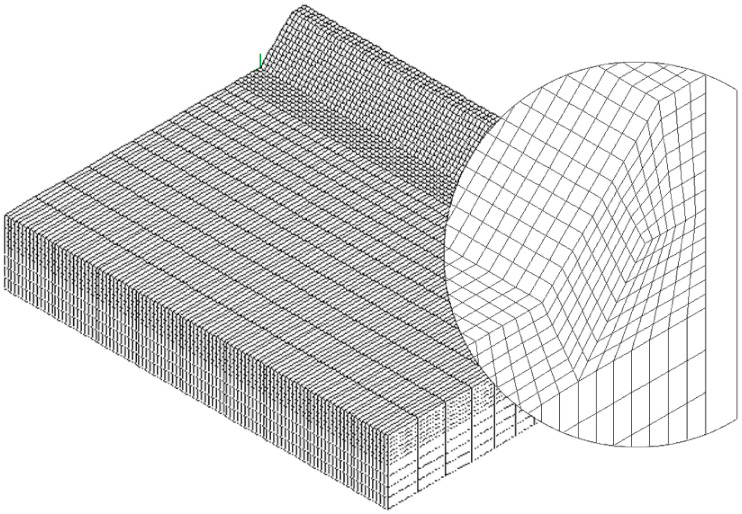	0.28 #FE 47040 #nodes 45792	251	153.01 (half-width longitudinal section)
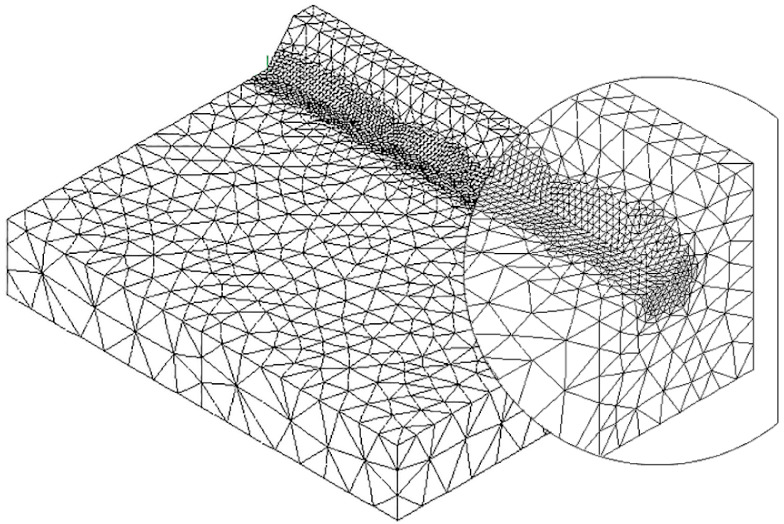	0.25 #FE 73250 #nodes 97041	331	151.36 (half-width longitudinal section)

**Table 3:** Comparison of FE mesh patterns adopted to estimate the R-NSIF K_I_ according to the Peak Stress Method (PSM).

Element Type	Element Size d (mm)	FE Mesh
4-node plane	0.28	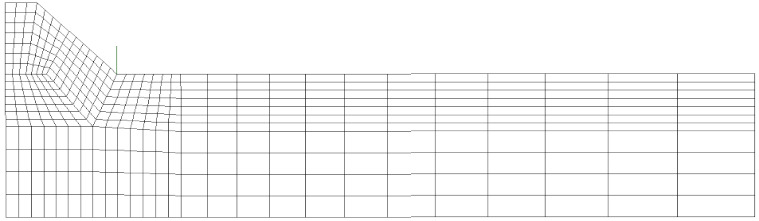
8-node brick	0.28	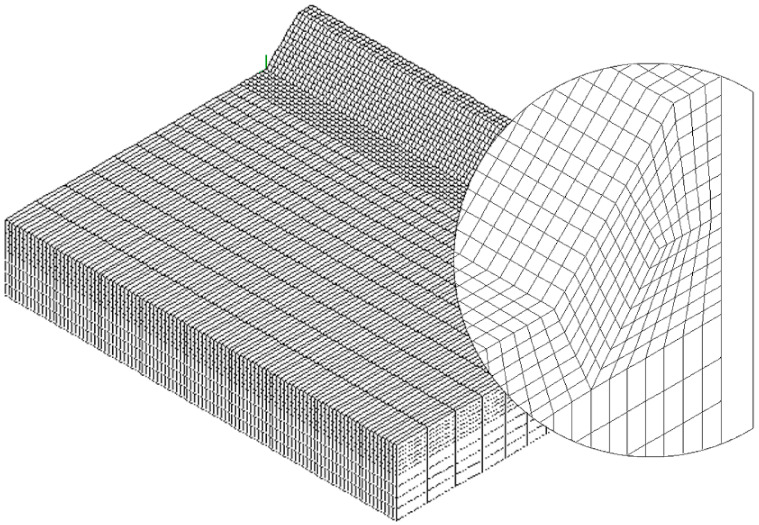
10-node tetra	0.25	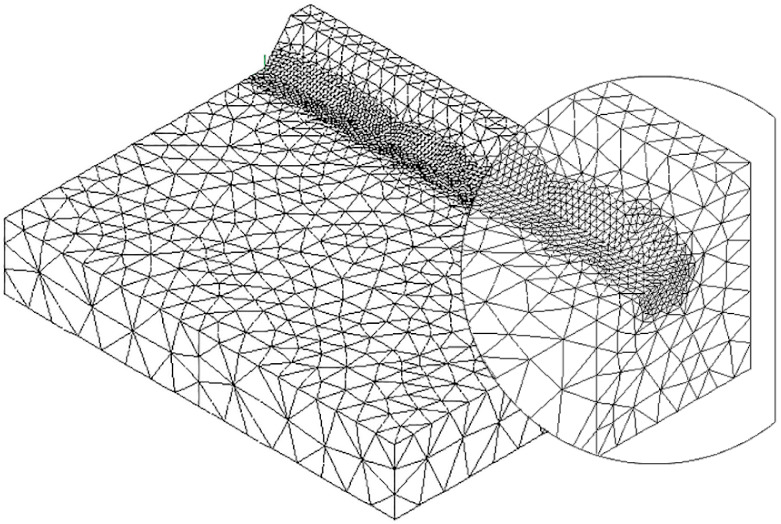
	0.33	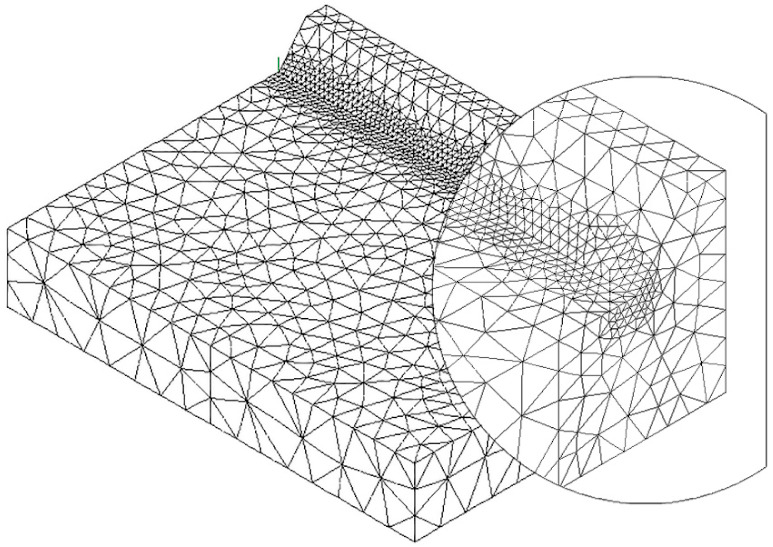
	1	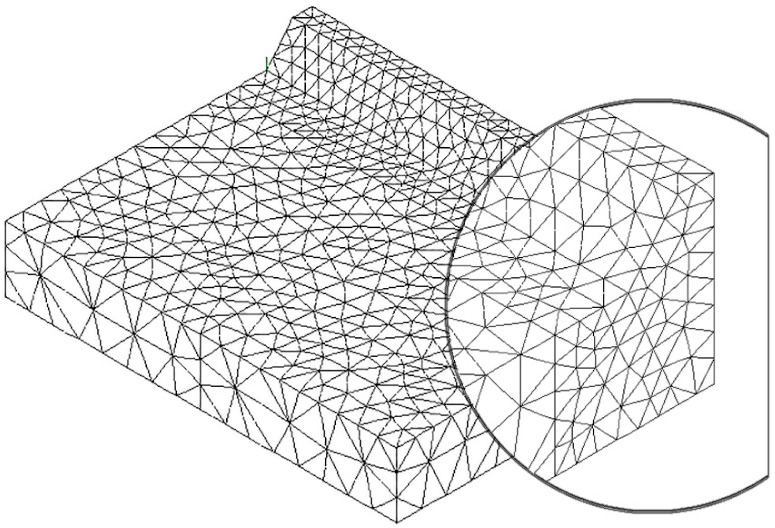

## Data Availability

Data is contained within the present article. Other data presented in this study are available in [40,41,42].

## Nomenclature

a_f_	Gaussian parameter of Goldak’s heat source	(mm)
a_r_	Gaussian parameter of Goldak’s heat source	(mm)
b	Gaussian parameter of Goldak’s heat source	(mm)
c	Gaussian parameter of Goldak’s heat source	(mm)
d	average element size adopted to apply PSM	(mm)
K^*^_FE_	calibrated coefficient to apply PSM	
K_I_	NSIF or R-NSIF under mode I	(MPa mm^1−λI^)
Q_f_	maximum power density of the frontal source	(W/mm^3^)
Q_F_	frontal power density	(W/mm^3^)
Q_r_	maximum rear power density	(W/mm^3^)
Q_R_	rear power density	(W/mm^3^)
r	radial coordinate	(mm)
θ	angular coordinate	
t	time	(s)
T	temperature	(°C)
v	welding speed	(mm/s)
2α	notch opening angle	(°)
ε_r_	emissivity	
λ_I_	stress singularity under mode I	
σ_I,peak_	opening peak stress to apply PSM	(MPa)
σ_r_	Stefan–Boltzmann constant	
σ_θθ_	Residual stress, θθ component	(MPa)
σ_rr_	Residual stress, rr component	(MPa)
